# The Tricarboxylic Acid Cycle, an Ancient Metabolic Network with a Novel Twist

**DOI:** 10.1371/journal.pone.0000690

**Published:** 2007-08-01

**Authors:** Ryan J. Mailloux, Robin Bériault, Joseph Lemire, Ranji Singh, Daniel R. Chénier, Robert D. Hamel, Vasu D. Appanna

**Affiliations:** Department of Chemistry and Biochemistry, Laurentian University, Sudbury, Ontario, Canada; University of Texas-Houston Medical School, United States of America

## Abstract

The tricarboxylic acid (TCA) cycle is an essential metabolic network in all oxidative organisms and provides precursors for anabolic processes and reducing factors (NADH and FADH_2_) that drive the generation of energy. Here, we show that this metabolic network is also an integral part of the oxidative defence machinery in living organisms and α-ketoglutarate (KG) is a key participant in the detoxification of reactive oxygen species (ROS). Its utilization as an anti-oxidant can effectively diminish ROS and curtail the formation of NADH, a situation that further impedes the release of ROS via oxidative phosphorylation. Thus, the increased production of KG mediated by NADP-dependent isocitrate dehydrogenase (NADP-ICDH) and its decreased utilization via the TCA cycle confer a unique strategy to modulate the cellular redox environment. Activities of α-ketoglutarate dehydrogenase (KGDH), NAD-dependent isocitrate dehydrogenase (NAD-ICDH), and succinate dehydrogenase (SDH) were sharply diminished in the cellular systems exposed to conditions conducive to oxidative stress. These findings uncover an intricate link between TCA cycle and ROS homeostasis and may help explain the ineffective TCA cycle that characterizes various pathological conditions and ageing.

## Introduction

All aerobic organisms rely predominantly on the TCA cycle to generate NADH and FADH_2_ from acetyl CoA, a product obtained by the decarboxylation of pyruvate [1], [2]. NADH provides the bulk of the reductive potential necessary for the synthesis of ATP during oxidative phosphorylation. The reduced nicotinamide nucleotide is oxidized by complex I and the electrons (e^−^) are subsequently transferred to complex III and complex IV where they convert O_2_ into H_2_O [3]. The proton motive force created by the movement of e^−^ is harnessed to produce ATP with the participation of ATP synthase. Although this process is universal among all aerobic organisms, it is inherently dangerous due to its ability to create a highly oxidative intracellular environment. The inefficient transfer of e^−^ via the respiratory complexes results in the one electron reduction of diatomic oxygen, a phenomenon known to generate toxic ROS. Proteins, lipids, and nucleic acids are potent targets of these reactive moieties with disastrous consequences [4].

ROS are the effectors of a plethora of biochemical events and are known to be the causative agents in numerous diseases [5], [6]. Such pathological conditions as Alzheimer's disease, Amyotrophic Lateral Sclerosis, and cancers are apparently triggered by abnormal levels of ROS [7], [8]. As aerobic organisms are constantly faced with the dangers of ROS, it is not surprising that they have evolved intricate antioxidant strategies to combat these toxic molecules [9]. Catalase (CAT), superoxide dismutase (SOD), and glutathione peroxidase are among the critical enzymes that are utilized to keep ROS in check. The tripeptide glutathione is also critical in the detoxification of ROS [10]. These systems depend on reduced nicotinamide dinucleotide phosphate (NADPH) for their biological functions [11]. Metabolic networks and enzymes such as glucose-6-phosphate dehydrogenase (G6PDH), malic enzyme(ME), and NADP-ICDH that contribute in the production of NADPH are also key to the survival of aerobic organisms exposed to oxidative tension [12].

Although oxidative defence mechanisms are an integral component of all aerobic organisms, it is crucial to evaluate if the TCA cycle, the main producer of the pro-oxidant NADH, can modulate intracellular ROS production. A diminution of the TCA cycle will indeed decrease NADH production and consequently limit the oxidative danger posed by the oxidation of NADH and subsequent release of e^−^. In this report, using *Pseudomonas fluorescens* and the HepG2 cell line as model systems, we show how the TCA cycle is modulated with the aim of controlling the intracellular concentration of ROS. Exposure of these aerobic systems to ROS-producing metals and compounds perturbed the activity of several key TCA cycle enzymes promoting the accumulation of KG. The significance of KG, a metabolite that can detoxify H_2_O_2_ and O_2_^−^ with the concomitant formation of succinate in this process is also discussed.

## Results

*P. fluorescens* and HepG2 cells were challenged respectively with menadione (O_2_^−^ producer), hydrogen peroxide, and various toxic metals known to promote oxidative stress. Following treatment of these cells with these toxicants, oxidized lipids and proteins in *P. fluorescens* and HepG2 cells was analyzed. Marked increments in oxidized lipids and proteins were evident in *P.fluorescens* and HepG2 cells exposed to Al, Ga, Zn, and menadione compared to controls (Table 1 and Table 2). The oxidative properties of these metals were also confirmed by measuring H_2_O_2_ formation. Indeed the cell free extract (CFE) isolated from *P.fluorescens* exposed to Al, Ga, or menadione produced more H_2_O_2_ when incubated in 5 mM citrate (Table 3). Similar experiments were performed with HepG2 cells exposed to Al. Dichlorofluorescein-diacetate analysis revealed a higher level of intracellular ROS in HepG2 cells exposed to Al (data not shown). Following the establishment of the oxidative damage suffered by the cells exposed to these toxicants, it was important to evaluate how cellular metabolism was affected under these conditions. *P. fluorescens* and HepG2 cells exposed to metal toxicants and ROS-producing molecules accumulated succinate in the media, a biomarker for oxidative stress (data not shown) [13]. To probe the disparate metabolic profiles observed during oxidative stress further, cell-free extracts from *P. fluorescens* and HepG2 cells exposed to labelled citrate were analyzed by ^13^C-NMR and HPLC.

**Table 1:** Oxidized lipid and protein profile in *P. fluorescens* exposed to oxidative and metal stress

	Oxidized lipids (nmol MD/mg protein)	Oxidized protein (pmol carobonyl/mg protein
Control	0.241±0.040	0.025±0.002
A1-stressed	0.510±0.010	0.050±0.002
Ga-stressed	0.454±0.012	0.037±0.015
menadione	0.671±0.040	0.075±0.003

n = 3±S.D., p≤0.05

**Table 2:** Oxidized lipid and protein profile in HepG2 cells exposed to metal stress

	Oxidized lipids (µmol MDA/4×10^6 ^cells)	Oxidized protein (pmol carobonyl/4×10^6 ^cells)
Control	0.024±0.001	0.465±0.002
A1-stress	0.609±0.017	1.302±0.007
Zn-stress	0.156±0.093	1.209±0.058

n = 3±S.D., p≤0.05

**Table 3:** H_2_0_2 _concentrations in *P. fluorescens* exposed to oxidative and metal stress

	H_2_0_2 _concentration (µmol/mg protein)
Control	9.501±0.301
A1-stress	19.92±0.098
Ga-stress	33.32±1.298
Menadione	40.23±0.381

n = 3±S.D., p≤0.05

2 mg/ml equivalent of CFE was incubated in a reaction buffer containing peroxidase, p-anisidine, and 5 mM citrate. The reaction was stopped after 30min and the amount of H_2_0_2 _was quantified.

**Figure 1:**
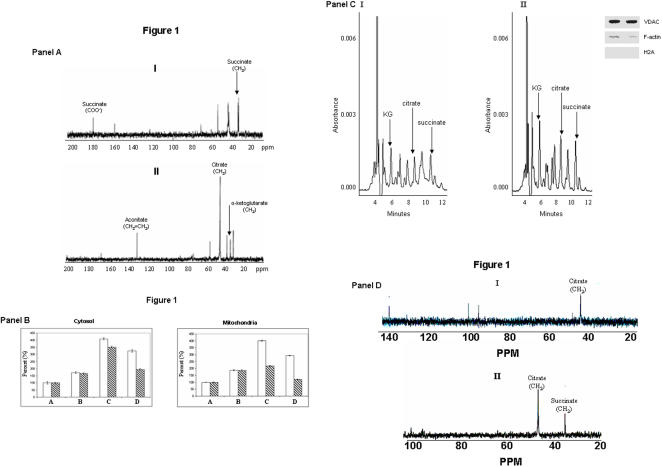
Metabolite analyses in *Pseudomonas fluorescens* and in HepG2 cells. Panel A: ^13^C-NMR analyses of CFE from *Pseudomonas fluorescens* grown in a defined medium with citrate as the sole carbon source. I) Ga-^13^C_2_-2,4-citrate and NAD as substrates II)^13^C_2_-2,4-citrate and NAD as substrates **Panel B**: Analysis of α-ketoglutarate (open bars) and succinate (crossed bars) in the cytosol and mitochondria of HepG2 cells exposed to control (**A**), Al-citrate (**B**), Al-treated cells exposed to 5 mM KG (**C**), and control cells exposed to Al-citrate (**D**). Samples were treated treated with perchloric acid and injected into the HPLC. The peaks were manually quantified using EMPOWER software. 100% corresponds to the absorbance value for KG and succinate peaks in the control cytosol and mitochondria (cytosol: 100% for KG and succinate is equivalent to an absorbance value of 0.0015 and 0.0012. Mitochondria: 100% for KG and succinate is equivalent to an absorbance value of 0.002 and 0.003). n = 3, mean±S.D., p≤0.05. **Panel C**: Representative chromatographs showing the consumption of citrate in HepG2 mitochondria. Mitochondria were isolated following a 24 h exposure to **I)** citrate and **II)** Al-citrate. Mitochondria were incubated for 1h at 37°C in a phosphate reaction buffer consisting of 1 mM citrate and 0.1 mM NAD. Top right corner: the purity of the mitochondrial fraction was assessed by the immunodetection of VDAC, F-actin, and H2A (note: for all NMR and HPLC experiments the purity of the fractions was assessed prior to the experiment). **Panel D**: Proton-decoupled ^13^C-NMR spectra obtained from the incubation of whole HepG2 cells with 10 mM ^13^C_2_-2,4-citrate for 24 h. HepG2 cells were exposed to **I)** citrate and **II)** Al-citrate for 24h and isolated for the ^13^C-NMR analysis.

**Figure 2:**
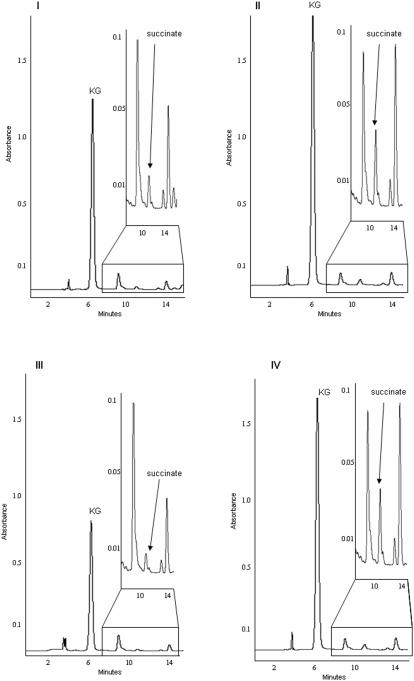
Analysis of the KG mediated detoxification of H_2_O_2_. 1 mg/ml equivalent of membraneous protein from control and Al-stressed *P. fluorescens* was reacted for 15min in the following conditions: I) control protein+5 mM KG+5 mM H_2_O_2_, II) Al-stressed protein+5 mM KG+5 mM H_2_O_2_, III) Al-stressed protein+5 mM KG+5 mM H_2_O_2_+10units of catalase, and IV) control protein+5 mM KG+5 mM H2O2+50 µM malonate+50 µM NaN3. The reaction mixtures were then treated accordingly for HPLC. Time zero measurements and reactions in the absence of protein were performed to assure the peak specificity.

The first evidence for the intriguing role of the TCA cycle in modulating oxidative tension was obtained when Ga-citrate was incubated with the CFE (cell-free extract) from *P. fluorescens*. ^13^C-NMR chemical shifts at 32 ppm and 181 ppm attributable to the CH_2_ and COO^−^ of succinate were evident (Figure 1A). On the other hand, the diagnostic fingerprints indicative of KG were present in the CFE with citrate as the substrate (Figure 1A). No succinate peaks were evident. As NAD was the only exogenous cofactor utilized, KG was an important metabolite generated via the enzyme ICDH. However, in the presence of either Al or Ga, two metals known to generate ROS [14]–[16], succinate was also produced. The inclusion of catalase prior to the addition of the metal-citrate complex provided KG peaks only. The labelling pattern of ^13^C peaks would eliminate the production of succinate via isocitrate lyase (ICL). If this enzyme was involved, only a peak at 32 ppm indicative of the CH_2_ would have been present. Furthermore, the same diagnostic peaks were obtained in the presence of malonate (5 mM), a potent inhibitor of ICL (Figure S1). Thus, it appears that succinate was a product of the decomposition of KG by the ROS generated by Ga. Similarly, cells obtained from the Al and menadione media respectively did readily yield the succinate signal upon incubation with labelled citrate (Figure S2). Hence, the ^13^C-NMR data pointed to a metabolic shift promoting the detoxification of ROS in *P. fluorescens* subjected to Al, Ga or menadione. Studies performed with HepG2 cells exposed to Al, a pro-oxidant, also revealed the accumulation of KG and succinate. HPLC analyses of the control and Al-stressed HepG2 cells revealed the marked accumulation of both metabolites in cytosol and mitochondria of the Al-treated cells (Figure 1, Panel B). Treatment of control cells with Al-citrate for 24h confirmed the observed accumulation of KG and succinate during oxidative tension. In addition treatment of Al-stressed HepG2 cells with 5 mM KG for 24h encouraged the cytosolic and mitochondrial accumulation of succinate (Figure 1, Panel B). Thus these observations indicate that the oxidative insult provoked by Al toxicity encouraged the accumulation of KG and succinate, an end product of KG-mediated detoxification of ROS. To further confirm the mitochondrial accumulation of KG and succinate in Al-treated cells, mitochondria were treated for 1h with citrate and NAD. The mitochondria from the Al-stressed cells accumulated more KG and succinate following citrate treatment as opposed to control mitochondria (Figure 1, Panel C). In addition exposure of Al-stressed HepG2 cells with 10 mM ^13^C-labelled citrate confirmed the observed accumulation of succinate (Figure 1, Panel D). To confirm the antioxidant properties of KG, membrane fractions from control and Al-stress *P. fluorescens* were incubated in KG and H_2_O_2_. In contrast to the control fractions KG was poorly metabolized in the reaction mixture containing Al-treated membranes and the KG was strictly dedicated to the detoxification of H_2_O_2_ as indicated by the presence of a succinate peak (Figure 2). The inclusion of catalase in the Al-stressed reaction mixture seemed to ablate the antioxidant properties of KG as indicated by the lowered succinate signal (Figure 2). Thus, it became obvious that KG was an important component of the ROS detoxification strategy in these systems.

**Figure 3:**
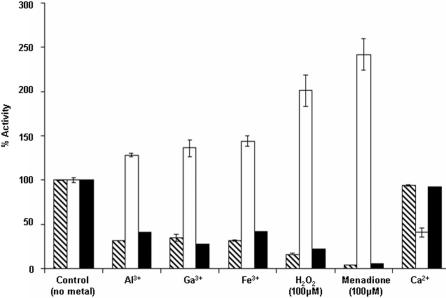
Activities of KGDH, NADP-ICDH, and NAD-ICDH in *P. fluorescens* grown in various media. KGDH activity was determined by incubating the membrane CFE (0.2 mg/mL protein equivalents) with 0.3 mM KG, 0.5 mM Coenzyme A and 0.5 mM NAD. Dinitrophenylhydrazone was monitored [38]. NADP-ICDH activity was determined by incubating soluble CFE (0.2 mg/mL protein equivalents) with 2 mM isocitrate, 4 mM malonate and 0.5 mM NADP. Formation of NADPH was monitored at 340 nm (ε = 6220 M-1 cm-1). For NAD-ICDH activity, the membrane CFE was utilized and NAD was the cofactor. In some instances, activities of these enzymes were also obtained by quantifying activity bands using Scion Imaging Software for Windows (SCION corperation, Frederick, MD). All activities are expressed as a percent of the control (100% corresponds to corresponds to 40 nmol.min-1.mg protein-1 for KGDH, 55 nmol.min-1.mg protein-1 for NAD-ICDH, and 55 nmol.min-1.mg protein-1 for NADP-ICDH). Crossed bar: KGDH, open bar: NADP-ICDH, and solid bar: NAD-ICDH. Values are mean±SD, n = 3, p≤0.05.

These findings prompted us to probe the activity and expression of the key enzymes involved in the homeostasis of this keto acid, namely KGDH, NADP-ICDH, and NAD-ICDH. When *P. fluorescens* was exposed to Ga, Al, Fe, H_2_O_2_ or menadione, all known to create an oxidative environment, the activity of NADP-ICDH was increased while the activities of KGDH and NAD-ICDH were markedly decreased. Compared to the controls, a 3-fold reduction in KGDH activity was observed in a Ga-stressed medium. However in a Ca-citrate culture, a metal not known to perturb the redox environment, the activity of this enzyme was similar to that observed in the control cultures (Figure 3). Similarly, NADP-ICDH activity was higher in a menadione medium. At least a 2-fold increase compared to the control was recorded (Figure 3). This situation was reversed when these cells were transferred to a control medium (Figure S3). Irrespective of the source of carbon, this NADPH-generating enzyme was more active while the NADH producing counterpart and KGDH were less active in the cells subjected to an oxidative stress.

**Figure 4:**
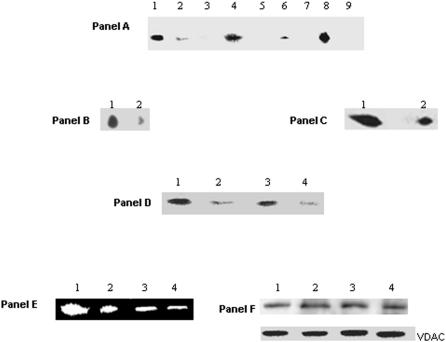
In-gel activity staining of KGDH. Panel A: Membrane CFE from *P. fluorescens* grown in various conditions (1–9) were analyzed. *Lane 1*: citrate medium, *Lane 2*: Ga-stressed medium, *Lane 3*: Al-stressed medium, *Lane 4*: malate medium; *Lane 5*: Al-malate medium; *Lane 6*: H_2_O_2_-stressed medium, *Lane 7*: O_2_^−^-stressed medium, *Lane 8*: glucose medium, *Lane 9*: glucose/H_2_O_2_-stressed medium. Panel B: 2D BN-PAGE activity staining of KGDH. *Lane 1*: membrane CFE from *P. fluorescens* grown in citrate medium. *Lane 2*: in Ga-citrate medium. Panel C: The bands from lanes 1 and 2 in Panel A were excised and analyzed by 2D immunoblot. *Lane 1*: citrate medium. *Lane 2*: Ga-citrate. Panel D: Modulation of KGDH activity in membrane CFE isolated from *P. fluorescens* grown in different media. *Lane 1*: *P. fluorescens* grown in citrate medium. *Lane 2*: Ga-citrate medium, *Lane 3*: Al-stressed cells introduced into citrate medium, *Lane 4*: citrate cells introduced into Al-citrate medium. Panel E: In-gel detection of mitochondrial KGDH in HepG2 cells. *Lane 1*: citrate, *Lane 2*: Al-citrate, *Lane 3*: Zn-citrate, and *Lane 4*: citrate and H_2_O_2_. Panel F: Immunoblot analysis of KGDH expression in HepG2 mitochondria. *Lane 1*: citrate, *Lane 2*: Al-citrate, *Lane 3*: Zn-citrate, and *Lane 4*: citrate and H_2_O_2_.

**Figure 5:**
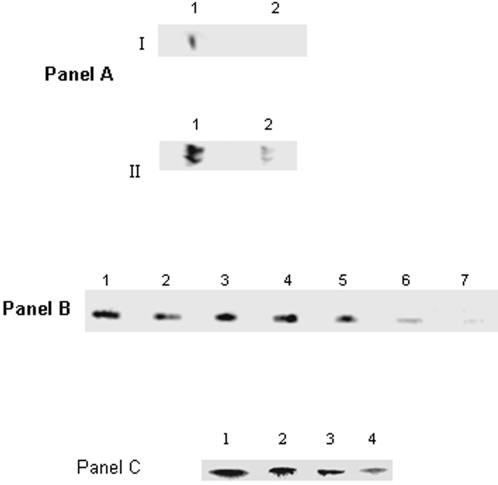
Activity and expression of NAD-ICDH in *P. fluorescens* membrane CFE and HepG2 mitochondria. Panel A: I) 2D PAGE activity stain for NAD-ICDH in *P. fluorescens* membrane CFE from *Lane 1*: citrate medium and *Lane 2*: Al-citrate medium. II) Activity bands were excised and detected by silver staining. *Lane 1*: citrate and *Lane 2*: Al-citrate. Panel B: In-gel NAD-ICDH activity in membrane CFE isolated from *P. fluorescens* grown in different media. *Lane 1*: citrate medium, *Lane 2*: Fe-citrate medium, *Lanes 3-7*: citrate media containing 0.1, 1.0, 5.0, 10.0 and 15.0 mM Al, respectively. Panel C: NAD-ICDH activity in the mitochondrial extract from HepG2 cells grown in *Lane 1*: citrate, *Lane 2*: Al-citrate, *Lane 3*: Zn-citrate, and *Lane 4*: H_2_O_2_ and citrate conditions.

**Figure 6:**
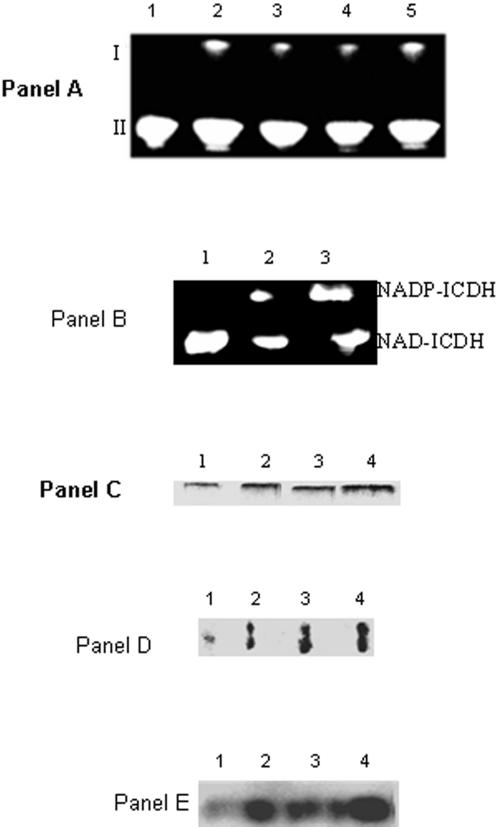
The detection of NADP-ICDH/NAD-ICDH in the soluble and the membrane CFE from *P. fluorescens* and the mitochondria from HepG2 cells. Panel A: BN-PAGE analysis of NADP-ICDH activity in soluble CFE isolated from *P. fluorescens* grown in various media. *Lane 1*: citrate, *Lane 2*: cells grown in Ga-citrate, *Lane 3*: control cells transferred into Ga-citrate media for 6 h, *Lane 4*: control cells transferred to medium with 1 mM menadione for 6h, and *Lane 5*: control cells transferred to medium with H_2_O_2_ (15 mM). I and II correspond to two bands with ICDH activities. Panel B: The membrane CFE isolated *P. fluorescens* grown in *Lane 1*: citrate, *Lane 2*: Al-citrate, and *Lane 3*: Ga-citrate media were tested for membrane NADP-ICDH and NAD-ICDH activity. Following the visualization of the NAD-ICDH activity band, the gel was treated with 2 mM isocitrate and 0.1 mM NADP. Panel C: HepG2 cells grown in *Lane 1*: citrate, *Lane 2*: Al-citrate, *Lane 3*: Zn-citrate, and *Lane 4*: citrate and H_2_O_2_ were analyzed for the presence of a mitochondrial NADP-ICDH. Following the detection of NAD-ICDH, NADP-ICDH bands were detected as in Panel A. Panel D: The NADP-ICDH activity bands from Panel C were excised and subject to 2D SDS-PAGE. *Lane 1*: citrate, *Lane 2*: Al-citrate, *Lane 3*: Zn-citrate, and *Lane 4*: citrate and H_2_O_2_. Panel E: 2D immunodetection of NADP-ICDH in HepG2 mitochondria isolated from HepG2 cells grown in *Lane 1*: citrate, *Lane 2*: Al-citrate, *Lane 3*: Zn-citrate, and *Lane 4*: citrate and H_2_O_2_ conditions.

**Figure 7:**
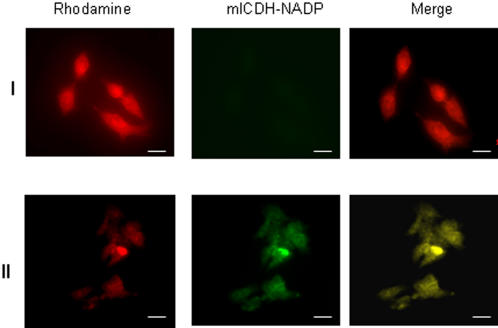
Immunofluorescent studies of mitochondrial NADP-ICDH in HepG2 cells subjected to oxidative stress. HepG2 cells were grown to a minimal density on coverslips and exposed to I) citrate and II) 0.5 mM Al-citrate for 24 h. The fluorescein isothiocyanate tagged secondary antibody (*green*) was used to visualize NADP-ICDH. The yellow fluorescence indicates the presence of NADP-ICDH in the mitochondria. Scale bar: 10 µm.

**Figure 8:**
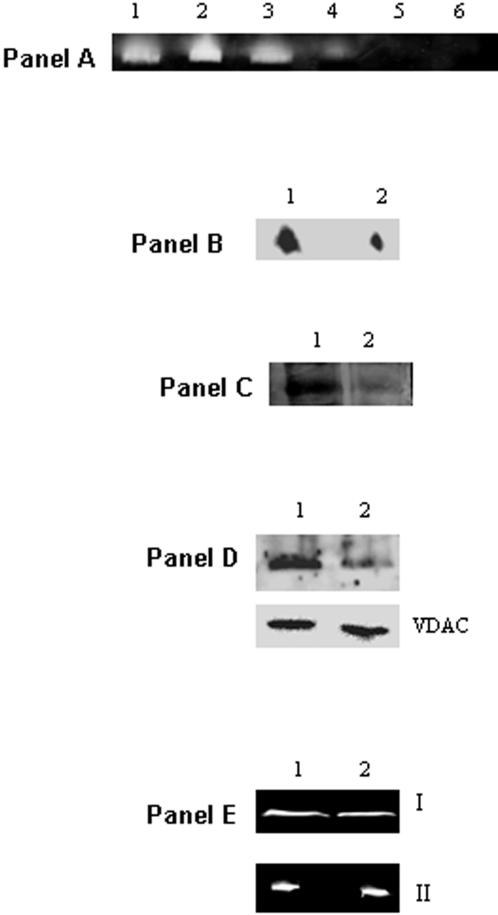
Determination of the activity and expression of SDH and MDH in *P. fluorescens* and the mitochondria of HepG2 cells. Panel A: In-gel detection of SDH activity and expression in *P. fluorescens* grown in *Lane 1*: citrate and *Lane 2-6*: citrate media containing 0.1, 0.5, 5, 10, and 15 mM Al. Panel B: 2D immunodetection of SDH in *P. fluorescens* grown in *Lane 1*: citrate and *Lane 2*: Al-citrate. Lanes 1 and 6 from panel A were excised and analyzed. Panel C: In-gel detection of SDH activity in mitochondria of HepG2 grown in *Lane 1*: citrate and *Lane 2*: Al-citrate conditions. Panel D: Immunodetection of SDH in the mitochondria of HepG2 cells grown in *Lane 1*: citrate and *Lane 2*: Al-citrate conditions. Panel E: In-gel detection of MDH activity in the mitochondria of HepG2 cells grown in *Lane 1*: citrate and *Lane 2*: Al-citrate conditions (I = activity stain, II = 2D electrophoresis).

Blue Native Polyacrylamide Gel Electrophoresis (BN-PAGE), 2D SDS-PAGE and immunoblot assays helped establish the relationship between activity and protein expressed. *P. fluorescens* grown in control, metal stress, and pro-oxidant media revealed the negative impact of the metal/oxidative stress on KGDH activity (Figure 4, Panel A). To establish if the TCA cycle was indeed an integral component of the cellular machinery involved in defending the organism against ROS, glucose and malate were utilized as the sole carbon sources respectively (Figure 4, Panel A). And, when the cells were exposed to oxidants like H_2_O_2_ and menadione, a significant decrease in KGDH activity was observed. The ability of a pro-oxidative environment to inhibit KGDH was further confirmed by two dimensional and immunoblot analysis of *P. fluorescens* grown in citrate or Ga-citrate containing media (Figure 4, Panel B and C). When Ga-stressed cells were introduced into citrate control media a significant increase in KGDH activity was observed (Figure 4, Panel D). Similarly a decrease in KGDH activity was evident upon the introduction of control cells into the Al containing media (Figure 4, Panel D). As KGDH is known to be a producer of ROS, its diminished activity will lead to a marked reduction of these oxidants [17]. These results strongly suggest that the TCA cycle is an important metabolic network utilized by organisms to survive an oxidative environment. In HepG2 cells, a decrease in KGDH activity was also observed, however there did not appear to be a significant change in expression of this dehydrogenase (Figure 4, Panels E and F). Since oxidative stress diminished the ability of KGDH to produce NADH, we decided to probe the activity and expression of other NADH producing enzymes in particular NAD-ICDH. NAD-ICDH displayed a marked decrease in *P. fluorescens* and HepG2 cells exposed to metal and oxidative stress (Figure 5). HPLC studies confirmed the alterations in nicotinamide dinucleotide metabolism as a result of oxidative stress. Bacteria and HepG2 cells exposed to menadione, Al, and Ga displayed higher NADP(H) and lower NAD(H) levels when compared to control cells (data not shown). The net impact of this concerted metabolic reconfiguration led to a dramatic decrease in NADH, a major contributor to the production of ROS *in vivo*. On the other hand, the overexpression of NADP-ICDH resulted in increased NADPH, a critical modulator of the cellular redox potential and KG for ROS detoxification (Figure 6). Indeed, increased activity and expression of the NADP-dependent enzyme was recorded in the soluble fraction from the bacterial cells (Figure 6, Panel A). The increased activity was attributed to the emergence of an isozyme at the upper part of the gel (Figure 6, Panel A represented by I). In addition increased NADP-ICDH activity was also recorded in the membrane components from *P. fluorescens* (Figure 6, Panel B). The mitochondrial extracts from the HepG2 cells exposed to oxidative stress also exhibited higher activity and expression of the NADP ICDH (Figure 6, Panel C-E). Immunofluorescence experiments confirmed the increased expression of the NADP-dependent mitochondrial ICDH in the HepG2 cells exposed to Al (Figure 7). Hence, elevated levels of NADPH, KG and decreased levels of NADH may contribute to the survival of an organism in an oxidative environment. Furthermore, the downstream enzyme succinate dehydrogenase (SDH) also displayed lowered activity and expression in the stressed conditions while malate dehydrogenase (MDH) did not show any significant change in activity (Figure 8).

## Discussion

These observations indicate that in an effort to circumvent oxidative stress, prokaryotic and eukaryotic cells upregulate NADP-ICDH to produce NADPH, a reducing equivalent required to regenerate antioxidants, and KG, a powerful antioxidant. In addition these data also provide novel insight into the role cytoplasmic and mitochondrial NADP-ICDH enzymes play in combating oxidative stress. Thus, the TCA cycle acts both as a scavenger and generator of ROS and its modulation appears to be an important strategy in O_2_-dependent organisms to regulate the intracellular levels of ROS. Hence, the role of this metabolic network may be more diverse than has been hitherto proposed. Unlike known ROS detoxifying agents like SOD and catalase that only decompose ROS without impacting on their production [18], the TCA cycle can both regulate their formation and participate in their decomposition. Hence, the upregulation of NADP-ICDH and the downregulation of KGDH and NAD-ICDH may enable an organism to control its redox status very effectively and the concomitant accumulation of succinate may act as a potent signal for anaerobic respiration.

The present study extends the importance of the TCA cycle beyond its classical role in oxidative phosphorylation and anabolic reactions to the homeostasis of ROS. It appears that the interplay between KG production and utilization constitutes an important strategy aimed at modulating the concentration of ROS. By decreasing the activity of KGDH, these cellular systems appear to dedicate KG to ROS scavenging with the concomitant reduction in NADH production. The KGDH with its lipoic acid localized in the E_2_ subunit would be a natural sensor of intracellular oxidative tension [19]. Its oxidation would signal the inactivation of its enzymatic activity and the channelling of KG towards the detoxification of ROS [20], [21]. In addition the ensuing decrease in NADH would result in less ROS formation. Hence, it is not surprising that in numerous disorders, a dramatic reduction in KGDH activity is observed [22], [23]. This may be an adaptive mechanism aimed at the detoxification of ROS, an important mediator of various pathological conditions [24]. Such a strategy would indeed limit ATP production but would protect the cell from the damage of ROS and extend its survivability, an overriding goal for most cells. The diminution of KGDH activity appears to be central in the regulation of the TCA cycle. This enzyme produces NADH, a prooxidant and is known to generate ROS [17]. Its decreased activity would undoubtedly alleviate the burden imposed during oxidative stress and increase the pool of KG, a scavenger of ROS. Thus, the modulation of KGDH appears to be a key component of the ROS detoxification strategy. It may control the production of NADH via the TCA cycle and also provide KG for combating oxidative stress. The role of metabolic enzymes in attenuating oxidative stress has recently been reported [24]–[26]. Hence, it is tempting to postulate that when given the choice between extending cellular longevity and producing ATP for other physiological functions, the cell will opt for the former.

KG is a central metabolite in numerous metabolic networks and provides a link between carbohydrate and protein metabolism. It is utilized in various hydroxylation reactions essential in oxygen sensing, DNA repair, and the synthesis of L-carnitine. Thus this keto acid may provide an interesting gauge of the oxidative status of a cell. Even though keto acids have been used to prevent inflammation and oxidative stress in a clinical setting, this is the first demonstration of the role of KG in the detoxification of ROS in both prokaryotes and eukaryotes. This is indeed a very effective tool in neutralizing ROS as it has a dual role of heralding the efficacy of the O_2_-mediated ATP-producing machinery in the cell. The succinate generated when ROS are detoxified by KG, may act as an intracellular mediator of anaerobiosis [27]. Indeed, succinate has been shown to activate HIF-1α due to its role in inhibiting the hydroxylation of proline [28], a reaction catalyzed by prolyl hydroxylase. It has recently been shown that KG can promote aerobic respiration and alleviate hypoxia [29].

**Figure 9:**
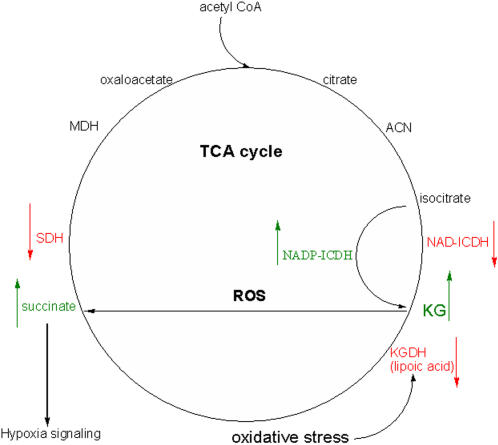
The TCA cycle and ROS homeostasis (KG is a potent ROS scavenger. Green = increase, Red = decrease).

The results reported here point to a novel biochemical function for the TCA cycle. Due to its ability to both produce and detoxify ROS, this metabolic network appears to be a very effective tool in modulating the redox status of a cell. KG serves the dual purpose of scavenging the ROS and consequently signalling anaerobiosis as a result of succinate production. Unlike other anti-oxidant defence mechanisms that are primarily targeted towards the detoxification of the oxidizing moieties, only the TCA cycle can both control their formation and participate in their decomposition. Furthermore, the resulting metabolites can also function as signalling molecules [30]. Figure 9 provides an overview of the role of the TCA cycle in modulating ROS production. Thus, an ineffective TCA cycle observed in numerous diseases may be an adaptive mechanism aimed at diminishing ROS production and extending cellular longevity.

## Methods

### Cell culture and isolation of cellular fractions

The bacterial strain *P. fluorescens* 13525 was obtained from American Type Culture Collection (ATCC) and ^13^C_2_-2,4 labelled citric acid was from Isotec, Cambridge.

*P. fluorescens* was grown in a mineral medium with citrate, the sole carbon source as described in [31]. The test metals (AlCl_3_; 15 mM, Ga(N0_3_); 1 mM, FeCl_3_; 0.1 mM, CaCl_2_; 1 mM) were complexed to citrate (19 mM) and the media that were dispensed in 200 mL amounts were inoculated with 1 mL of stationary-phase cells. Menadione (100 µM) and H_2_O_2_ (100 µM) were utilized for ROS stress. When malate or D-glucose was used as carbon sources their concentration was 19 mM. The Bradford protein assay was utilized to determine microbial growth [32]. HepG2 cells were a gift from Dr. D. Templeton (University of Toronto) and were maintained in α-minimal Eagle medium (α-MEM) supplemented with 5% fetal bovine serum (FBS) and 1% antibiotics. Cells were routinely seeded and cultured in 175 cm^2 ^flasks and incubated with 5% CO_2_ in a humidified atmosphere at 37°C. At 70% confluency, the cell cultures were re-supplemented with serum-free media containing citrate (2.5 mM), Al-citrate (0.5 mM∶2.5 mM), Zn-citrate (50 µM∶2.5 mM citrate) and H_2_O_2_-citrate (40 µM∶2.5 mM citrate) respectively and exposed from 4 hrs to 24 hrs. Cell viability was monitored with the aid of the Trypan Blue exclusion Assay. Following centrifugation, cells were suspended in a cell storage buffer (CSB, 50 mM Tris-HCl, 1 mM phenylmethylsulfonyl fluoride (PMSF), 1 mM dithiothreitol, 250 mM sucrose, 2 mM citrate, 1 mg/ml pepstatin, and 0.1 mg/mL leupeptin). Mitochondrial and soluble fractions were isolated by differential centrifugation [33]. To assess the purity of each fraction, immunblot was performed on the cytoplasm and the mitochondria to test for the presence of voltage dependent anion channel (VDAC), histone 2A (H2A), and F-actin

### Oxidized lipid and protein analysis and H_2_O_2_ measurement

The thiobarbituric acid reactive species assay (TBARS) was performed in order to evaluate the amount of oxidized lipids in the membrane, according to the method in [34]. Determination of protein carbonyl content was achieved with the dinitrophenyl hydrazine (DNPH) assay, as described in [35]. H_2_O_2 _production was assessed using the method described in [36]. 2 mg/ml equivalent of CFE was incubated in a reaction buffer (25 mM Tris, 5 mM MgCl_2_, pH 7.0) containing peroxidase, p-anisidine, and 5 mM citrate. The reaction was stopped after 30min and the absorbance was recorded at 458nm.

### Enzyme assays, electrophoretic analyses and immunoblot experiments

The CFE (50–200 µg protein equivalent) were incubated at room temperature in a 1 or 2 mL reaction mixture containing 25 mM Tris-HCl (pH 7.3), 5 mM MgCl_2_ with the respective substrates and cofactors (0.1 mM NAD, 0.1 mM NADP) for 10 min. The reaction was also monitored at 340nm and 450nm using 2, 4-dinitrophenylhydrazine [37], [38] respectively. KGDH activity was monitored in the membrane CFE using KG (0.3 mM), CoA (0.5 mM), NAD (0.5 mM). Controls were performed with reaction mixture devoid of the reactants.

BN-PAGE was performed according to a modified method developed by Schägger [39]–[41]. Enzymatic activity was visualized by the precipitation of formazan via phenazine methosulphate (PMS) and iodonitrotetrazolium (INT). To limit cross reaction NADP-ICDH and NAD-ICDH activities, the concentration of the cofactor ranged from 0.01–0.1 mM. Protein expression was analyzed by either Coomassie, silver stain, or immunoblot techniques following 2D BN-PAGE, 2D SDS-PAGE and 3D SDS-PAGE. Immunoblotting experiments employed antibodies directed against KGDH (E_2_ subunit), SDH, and NADP-ICDH were generously supplied by Dr. G. Lindsay (University of Glasgow), Dr. Lemire (University of Alberta), and Dr. S. Yokota (University of Yamanashi) respectively. The band intensities were quantitated using Scion Image for Windows (Scion Corporation, Frederick, Maryland, USA). 40–60 µg of protein was loaded into each well. For 2D and 3D SDS PAGE the activity bands from the in-gel activity stain were excised and treated with 1% β-mercaptoethanol and 1% SDS for 1h. The digested activity bands were then loaded into the gel and electrophoresed under denaturing conditions. Upon completion the gel slab was subject to Coomassie staining, silver staining, or immunoblotting. 2D BN PAGE was also performed on the activity bands. Activity bands were excised, loaded immediately into the Native gel, and then electrophoresed under Native conditions. Following completion the gel was subject to further activity staining or subject to silver staining.

### ^13^C-Nuclear Magnetic Resonance (NMR) spectroscopy and HPLC Analyses

^13^C-NMR analyses were performed using the Varian Gemini 2000 spectrometer operating at 50.31 MHz for ^13^C. Reactions were assayed in phosphate buffer (10 mM phosphate, 5 mM MgCl_2_, pH 7.3), 5 mM labelled ^13^C_2_-2,4 citric acid or complexed (1∶1) with the appropriate metal and 3 mg protein equivalent of CFE were utilized. Reactions were scanned for 2,000 or 20,000 transients and the resulting signals were referenced to standard metabolite spectra. Similarly, HepG2 whole cells were incubated for 1h at 37°C in a phosphate reaction containing 10 mM ^13^C_2_-2,4 citrate. For HPLC analysis of metabolite content, 2 mg/ml of cytosol and mitochondria isolated from HepG2 cells exposed to control or Al-stressed conditions was diluted to 5×10^−3^ mg and injected into the column. Fractions were treated with 0.5% perchlorate prior to injection. Similar experiments were performed with control HepG2 cells exposed to Al for 24 h and Al-treated HepG2 cells incubated in 5mM KG for 24 h. KG accumulation and metabolism was analyzed by incubating 2 mg/ml (protein equivalent) of HepG2 mitochondria or membrane CFE from *P. fluroescens* for 1h in a phosphate buffer containing 1 mM citrate with either 0.1 mM NAD or 0.1 mM NADP. For *P. fluorescens*, reactions a 1 mg equivalent of membrane fraction was incubated for 15 min in a phosphate reaction buffer containing 5 mM KG and 5 mM H_2_O_2_. Reactions were stopped on ice with perchloric acid and the supernatant was analyzed with a C_18_-reverse phase column (phenomenex). A mobile phase consisting of 20 mM KH_2_PO4 (pH 2.9) operating at a flow rate of 0.7ml/min at ambient temperature was utilized. All NMR signals and HPLC peaks were confirmed by spiking with known standards.

### Immunofluorescence microscopy

HepG2 cells were grown on glass coverslips in control and Al-stressed conditions as described above. The coverslips were washed thoroughly with PBS and 0.5 mM EDTA and exposed for 30min to rhodamine B (10 µg/ml in 2ml of α-MEM, 37°C). The coverslips were fixed for 10min in a methanol/acetic acid solution (3∶1 v/v) and subsequently incubated for 1h with 5% FBS dissolved in TTBS (1% Tween 20). Following the incubation in primary antibody (anti-ICDH 1/200 dilution) for 2h and secondary antibody (anti-rabbit FITC conjugate 1/1000 dilution) for 1h, the coverslips were washed extensively with TBS. Fluorescence was visualized using an inverted deconvoluting microscope operating at the appropriate emission and excitation wavelengths. The anti-rabbit FITC conjugate was purchased from Santa Cruz.

### Statistical analysis

Data were expressed as mean±SD. Statistical correlations of data were checked for significance using the student t test. Experiments were performed twice and in triplicate.

## Supporting Information

Figure S1 13C-NMR analysis of the CFE from Pseudomonas fluorescens grown in a defined medium containing Ga-citrate. 2 mg/ml protein equivalent of CFE was incubated for 1h in a phosphate reaction buffer containing 5 mM Ga-13C2-2,4-citrate, 5 mM malonate, and NAD. The reaction was stopped by boiling for 5min and the sample was treated accordingly for NMR analysis.
(2.43 MB TIF)

Figure S2 13C-NMR analysis of the CFE from Pseudomonas fluorescens grown a defined medium containing I) citrate, II) Al-citrate, and III) citrate and menadione. 2 mg/ml protein equivalent of CFE was incubated for 1 h in a phosphate reaction buffer containing I) 5 mM 13C2-2,4-citrate, II) 5 mM Al-13C2-2,4-citrate, and III) .5 mM 13C2-2,4-citrate. NAD was utilized as a cofactor. The reactions were stopped by boiling for 5min and the sample was treated accordingly for NMR analysis.
(21.61 MB TIF)

Figure S3 Regulation of cytosolic NADP-ICDH activity. The activity of NADP-ICDH was determined in Pseudomonas fluorescens grown in Lane 1: citrate (control), Lane 2: citrate and menadione, Lane 3: menadione-treated cells cultured in control media for 8h, and Lane 4: control cells cultured in menadione-containing media for 8h. I and II correspond to the two NADP-ICDH isozymes.
(3.12 MB TIF)
