# Supplementary figures and images for: The Tricarboxylic Acid Cycle, an Ancient Metabolic Network with a Novel Twist
Source: PLoS One. 2007 Aug 1;2(8):e690. doi: 10.1371/journal.pone.0000690 (PMC1930152; doi:10.1371/journal.pone.0000690)

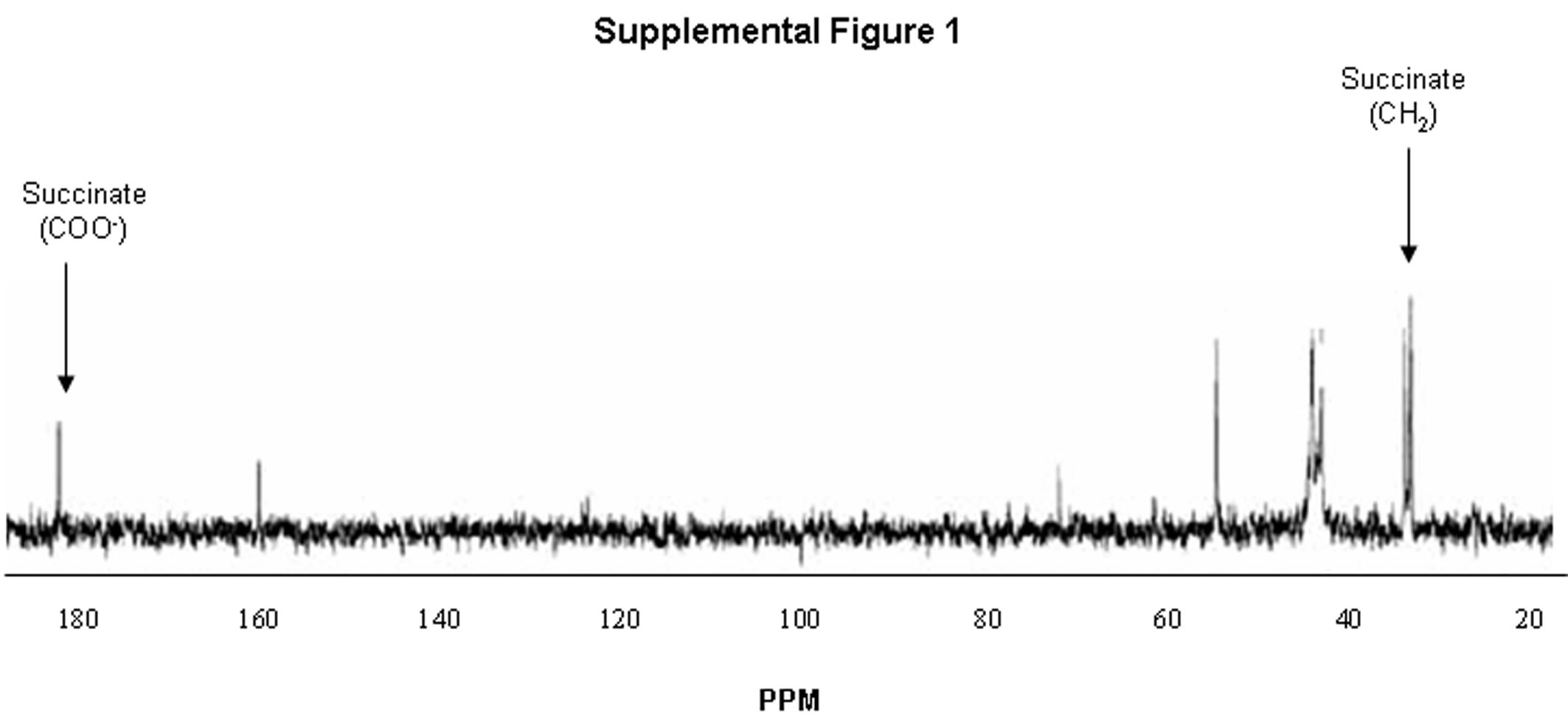

Supplement: Figure S1 — 13C-NMR analysis of the CFE from Pseudomonas fluorescens grown in a defined medium containing Ga-citrate. 2 mg/ml protein equivalent of CFE was incubated for 1h in a phosphate reaction buffer containing 5 mM Ga-13C2-2,4-citrate, 5 mM malonate, and NAD. The reaction was stopped by boiling for 5min and the sample was treated accordingly for NMR analysis. (2.43 MB TIF) [file pone.0000690.s001.tif]

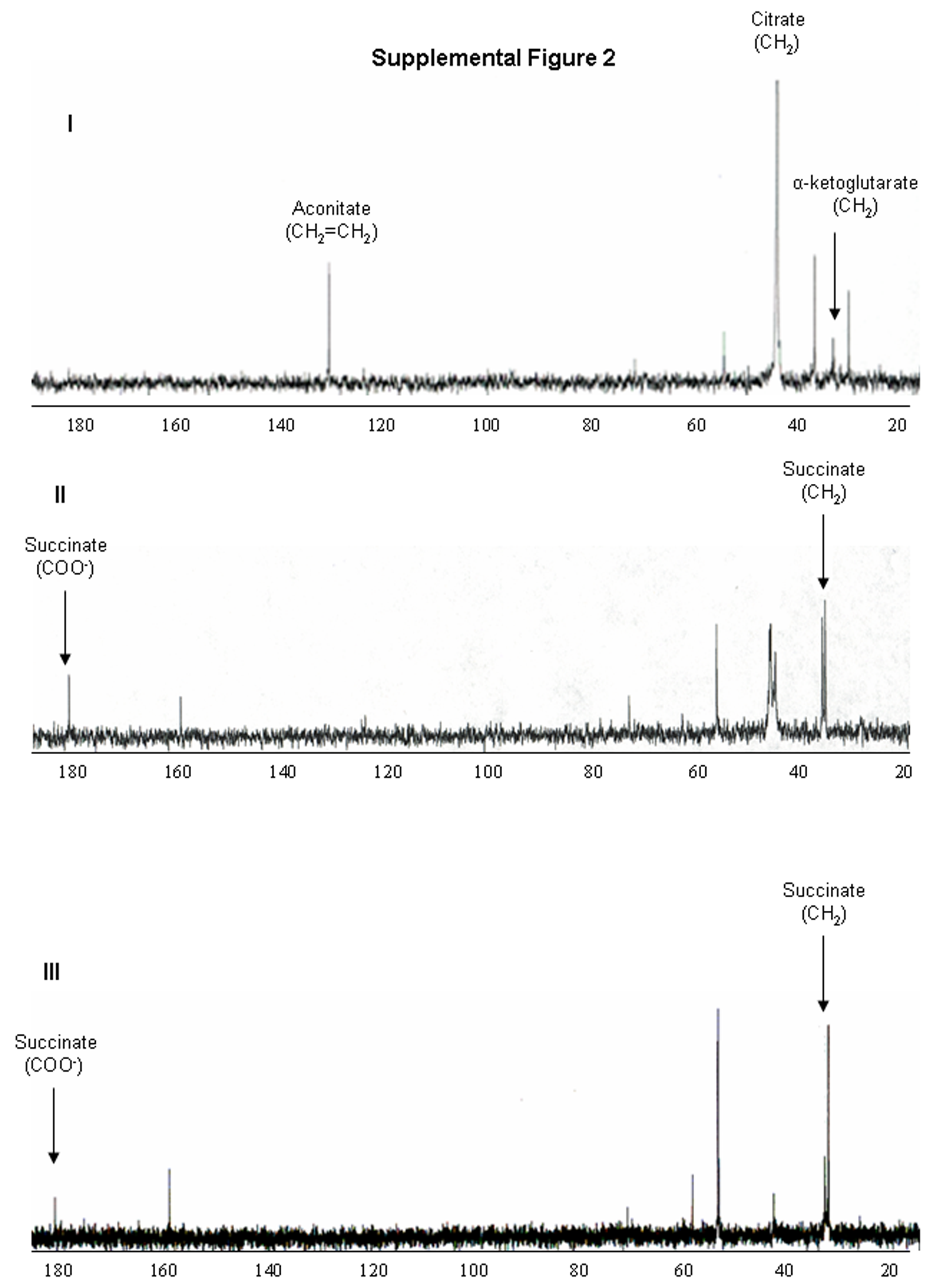

Supplement: Figure S2 — 13C-NMR analysis of the CFE from Pseudomonas fluorescens grown a defined medium containing I) citrate, II) Al-citrate, and III) citrate and menadione. 2 mg/ml protein equivalent of CFE was incubated for 1 h in a phosphate reaction buffer containing I) 5 mM 13C2-2,4-citrate, II) 5 mM Al-13C2-2,4-citrate, and III) .5 mM 13C2-2,4-citrate. NAD was utilized as a cofactor. The reactions were stopped by boiling for 5min and the sample was treated accordingly for NMR analysis. (21.61 MB TIF) [file pone.0000690.s002.tif]

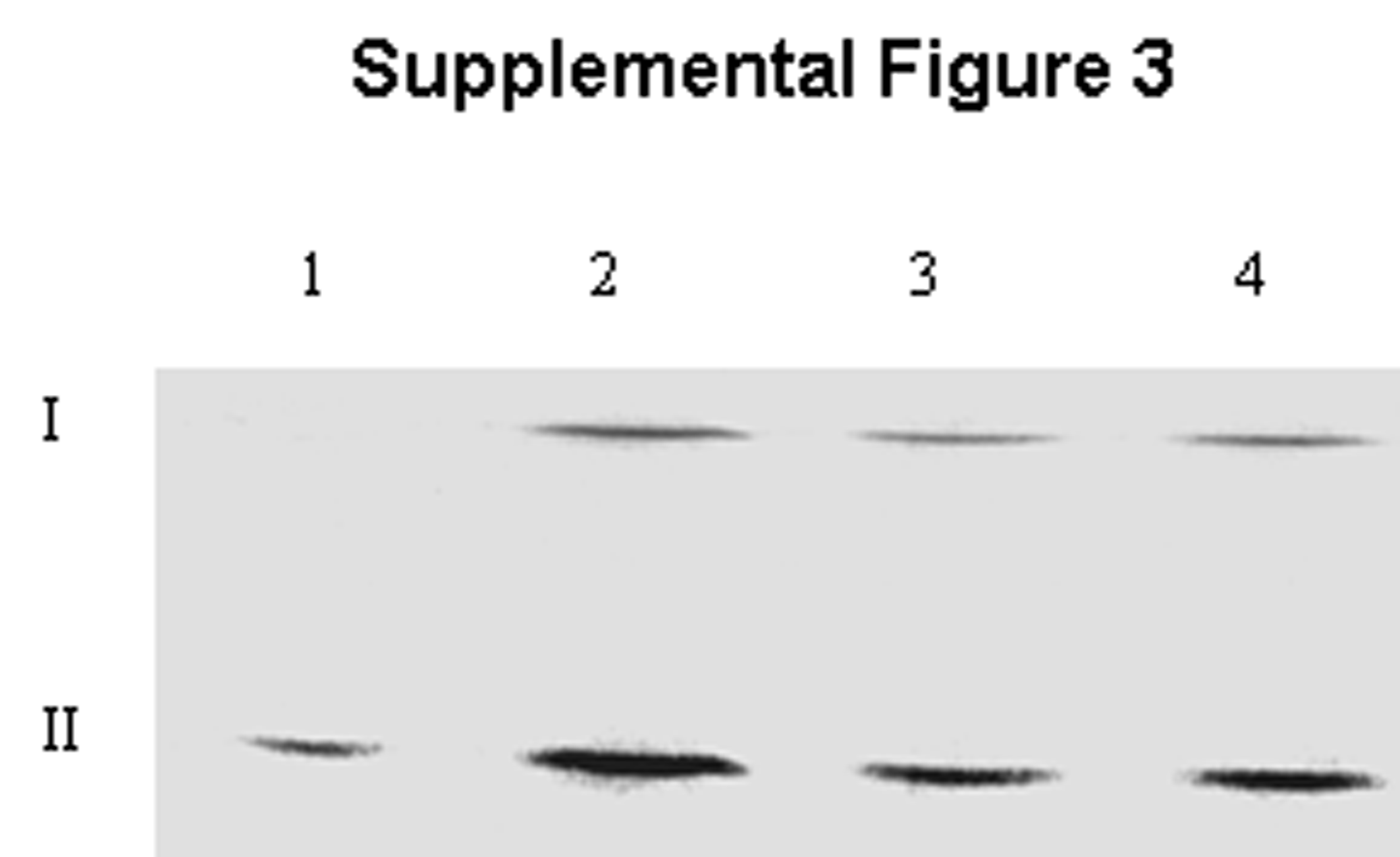

Supplement: Figure S3 — Regulation of cytosolic NADP-ICDH activity. The activity of NADP-ICDH was determined in Pseudomonas fluorescens grown in Lane 1: citrate (control), Lane 2: citrate and menadione, Lane 3: menadione-treated cells cultured in control media for 8h, and Lane 4: control cells cultured in menadione-containing media for 8h. I and II correspond to the two NADP-ICDH isozymes. (3.12 MB TIF) [file pone.0000690.s003.tif]
